# Simulation of supply chain disruptions considering establishments and power outages

**DOI:** 10.1371/journal.pone.0288062

**Published:** 2023-07-07

**Authors:** Hiroyasu Inoue, Yoshihiro Okumura, Tetsuya Torayashiki, Yasuyuki Todo

**Affiliations:** 1 Graduate School of Information Science, University of Hyogo, Kobe, Hyogo, Japan; 2 RIKEN Center for Computational Science, Kobe, Hyogo, Japan; 3 Faculty of Societal Safety Sciences, Kansai University, Suita, Osaka, Japan; 4 Disaster Reduction and Human Renovation Institution, Hyogo Earthquake Memorial 21st Century Research Institute, Kobe, Hyogo, Japan; 5 Research Institute of Economy, Trade and Industry, Chiyoda-ku, Tokyo, Japan; 6 Graduate School of Economics, Waseda University, Shinjuku-ku, Tokyo, Japan; Zhejiang Gongshang University, CHINA

## Abstract

In this paper, we simulate the economic loss resulting from supply chain disruptions triggered by the Great East Japan Earthquake (GEJE) in 2011, applying data from firm-level supply chains and establishment-level attributes to an agent-based model. To enhance the accuracy of the simulation, we extend data and models in previous studies in four ways. First, we identify the damage to production facilities in the disaster-hit regions more accurately by using establishment-level census and survey data and geographic information system (GIS) data on the damages caused by the GEJE and subsequent tsunami. Second, the use of establishment-level data enables us to capture supply chains between non-headquarter establishments in disaster-hit regions and establishments in other regions. Third, we incorporate the effect of power outages after the GEJE on production reduction, which exacerbated the effect of the supply chain disruption, particularly in the weeks immediately after the GEJE. Finally, our model incorporates sectoral heterogeneity by employing sector-specific parameters. Our findings indicate that the extended method can significantly improve the accuracy of predicting the domestic production after the GEJE, particularly due to the first three improvements utilizing various data sources, not because of the use of more sector-specific parameters. Our method can be applied to predict the economic effect of future disasters, such as the Nankai Trough earthquake, on each region more precisely.

## Introduction

The propagation of the effect of economic shocks through input-output linkages and supply chains resulting from natural disasters and other factors has been studied extensively [1]. Econometric studies using firm-level data have found that firms that are not directly affected by a disaster but linked to it through supply chains with directly affected firms tend to reduce their production, quantitatively confirming upstream and downstream propagation [2–5].

Other studies have used simulation approaches to address this issue. One approach involves applying input-output (IO) and computable general equilibrium (CGE) models to data at the region-industry level [6]. For example, several studies have used IO or CGE models to estimate the economic effects of the Great East Japan earthquake (GEJE) in 2011 and subsequent interindustry propagation [7, 8]. Other studies have employed extended CGE models to estimate the effect of fiscal policies after the GEJE [9, 10]. Additionally, some studies have employed similar IO/CGE approaches to examine the effect of the lockdowns of cities and regions to prevent the COVID-19 pandemic in 2020–22 [11, 12].

Another approach involves using agent-based models (ABMs) with firm-level data that include information on interfirm, rather than interindustry, supply chains. For example [13, 14], applied this approach to the case of the GEJE, estimating values of parameters in the model by using detailed data on domestic supply chains of approximately one million firms in Japan and production of Japan after the GEJE. This model was further applied to predict the effect of COVID-19 lockdowns on production [15, 16].

In comparing the two approaches, an advantage of using ABMs and firm-level supply chains is the ability to incorporate behaviors arising from the complexity of networks [17]. Thus, the dynamics of the propagation effect of economic shocks may be replicated more accurately. It is indeed shown that the propagation effect estimated from firm-level simulation is substantially larger than that from industry-level simulation possibly because of network complexity [13, 14]. Possible disadvantages of the use of ABMs is that they do not assume any optimization behavior and thus ignore responses to price changes and the optimal substitution of inputs across regions accommodated in CGE models [6]. However, when dealing with large-scale data, such as data for more than one million firms and their supply chains used in [13, 14], optimization assumptions are not feasible because of computational burdens.

Although the lack of optimization is inevitable in large-scale firm-level simulation, the previous analyses by [13, 14] have additional shortcomings. First, these studies rely on firm-level data without the location or transaction of each establishment, which makes it difficult to identify damage to non-headquarter establishments by the GEJE or supply chains between establishments. Second, while these studies capture damage to production facilities by the GEJE and the subsequent tsunami, they fail to incorporate production declines by electricity outages after the GEJE. These shortcomings may have led to underestimation of the initial shock of the GEJE and its propagation through establishment-level supply chains. Consequently, the previous studies [13, 14] fail to predict the actual production levels after the GEJE with precision, particularly the production immediately after the GEJE.

To achieve a better fit between the actual and simulated production of Japanese firms after the GEJE, this study expands the data, calibration method, and model used in [13, 14] in four ways. First, we incorporate data from an establishment-level survey in the disaster regions after the GEJE, the establishment-level census data in Japan, and detailed geographic information system (GIS) data on the earthquake intensity and the tsunami height. By combining these various data sources, we can identify damages to production facilities caused by the GEJE more accurately. The previous simulations [13, 14] and econometric estimations [2, 3, 5] that use only firm-level data may undervalue the economic shock of disasters because they cannot capture non-headquarter establishments hit by disasters. Second, using the establishment-level data also enables us to identify possible supply chains between non-headquarter establishments in the disaster regions and establishments in other regions that were ignored in the previous studies using firm-level data [13, 14]. Therefore, the simulation in this paper can estimate the propagation of the GEJE shock from the disaster regions to others more accurately than those in the previous studies that tend to undervalue the propagation. Third, in addition to damage to production facilities by the GEJE examined in [13, 14], we incorporate into our simulation the power outage after the GEJE that was caused directly by the disaster and indirectly by the accident at the Fukushima Daiichi Power Plant and exacerbated the effect of supply chain disruption. Accordingly, this research explains the large decline in production immediately after the GEJE that is not accurately predicted in the previous studies. Finally, our model employs more parameters by assuming sector-specific parameters.

We find that our extended method can greatly improve the accuracy of predicting the production of Japan after the GEJE. Moreover, our simulation results suggest that this improvement is mostly due to the first three improvements utilizing various data sources, not because of the use of more parameters in the model.

## Materials and methods

### Data

This section describes the data sources used in this study and explains how we combine them for our simulation analysis.

### Supply-chain data at the firm level

The main source of our analysis is data on Japanese firms collected by Tokyo Shoko Research (TSR), particularly its company information database and company linkage database. The former database contains attributes of each firm, including its address, industry classification, and sales, while the latter consists of its domestic clients and suppliers. We specifically use the data for 2011, the year of the GEJE. The number of firms in the data set is 1,161,096, and the number of supply chain links is 5,361,130. The data cover most firms in Japan, except for micro enterprises, and most major supply chain relationships between them.

Because sales of each supplier from each client and the final consumers are not available in the data, we estimate the transaction value between each firm pair by using the value between each sector pair taken from the 2015 input-output (IO) tables for Japan [18]. More precisely, we assume that sales of a firm to each of its clients are proportional to the client’s total sales, and that its sales to the final consumers are proportional to the firm’s total sales. Under these assumptions, the value of the transaction between each firm pair and between each firm and the final consumers is adjusted so that the sum of the inter-firm transaction values and the firm-to-consumer transaction values for each industry is the same as the production of the corresponding industry in the IO table. In this estimation process, we classify firms into 187 industries according to the IO tables, although in the simulation later, firms are classified into 1,460 industries according to the Japan Standard Industrial Classification [19]. Some firms are dropped from the sample because they lack total sales in the data. As a result, the number of firms in the sample is 966,627, whereas the number of links is 4,543,557.

#### Census data at the establishment level

Although the TSR data are quite useful in that they contain detailed supply-chain information on approximately one million firms, one shortcoming of the TSR data is that they do not include information on the location of establishments of each firm, except for its headquarters. Therefore, we utilize data from the Economic Census for Business Activity (hereafter the census) that are collected by the Ministry of Internal Affairs and Communications and the Ministry of Economy, Trade and Industry [20] and target all establishments in all industries in Japan, including establishments of micro-, small-, and medium-sized enterprises. We use the census data for 2016, where the number of establishments is 5,880,504 because matching the TSR data with the census data for 2016 turns out to be more successful than matching the TSR data with the previous census data for 2012. We merge the census data with the TSR data using firms’ names, addresses, and telephone numbers. As a result of this merging process, we add 1,014,673 non-headquarter establishments to the TSR data. Hereafter, we denote these combined data as the TSR-Census data.

The census data contain information on sales and the location of each establishment. Although the census data do not include any information on supply chains at the establishment level, we assume that any pair of establishments are linked through supply chains if they are linked at the firm level in the TSR data. Therefore, using the TSR-Census data enables us to specify damage to establishments in the disaster regions and to examine the propagation of its effect through supply chains between establishments more accurately.

#### Data on damage to production facilities and power outages

To identify the level of damage to production facilities at the establishment level, we additionally use data from an establishment-level survey conducted by the Research Institute of Economy, Trade and Industry one year after the GEJE titled “Questionnaire Survey on Damages to Companies Caused by the Great East Japan Earthquake” (hereafter the RIETI data) [21, 22]. The targets are 6,033 establishments in the manufacturing sector in the publicly defined disaster-hit regions, except for those in the regions most severely affected by the tsunami because of the difficulty of the implementation of the survey. The number of respondents was 2,117, a response rate of 35%. The RIETI survey asked each respondent about the level of damage to his or her production facilities on a scale of four levels: completely destroyed, half destroyed, partly destroyed, and not destroyed at all. The survey also asked how long each firm experienced a power outage after the GEJE. In the simulation, we utilize the information to determine the initial reduction rate of production capacity (100, 50, 25, and 0% if production facilities were completely, half, partly, and not destroyed, respectively) and the duration of production shutdowns of each firm included in the RIETI data.

#### Seismological and tsunami data

Because the number of establishments covered in the RIETI survey is far smaller than that in the TSR-Census data, we cannot identify the level of damage to production facilities and the duration of production shutdowns of all establishments in the TSR-Census data. Therefore, we estimate them by using the intensity of the GEJE at the city level from the seismological data of [23] and the height of the tsunami in each 100*m* × 100*m* mesh developed by [24] together with the RIETI data.

**Fig 1:**
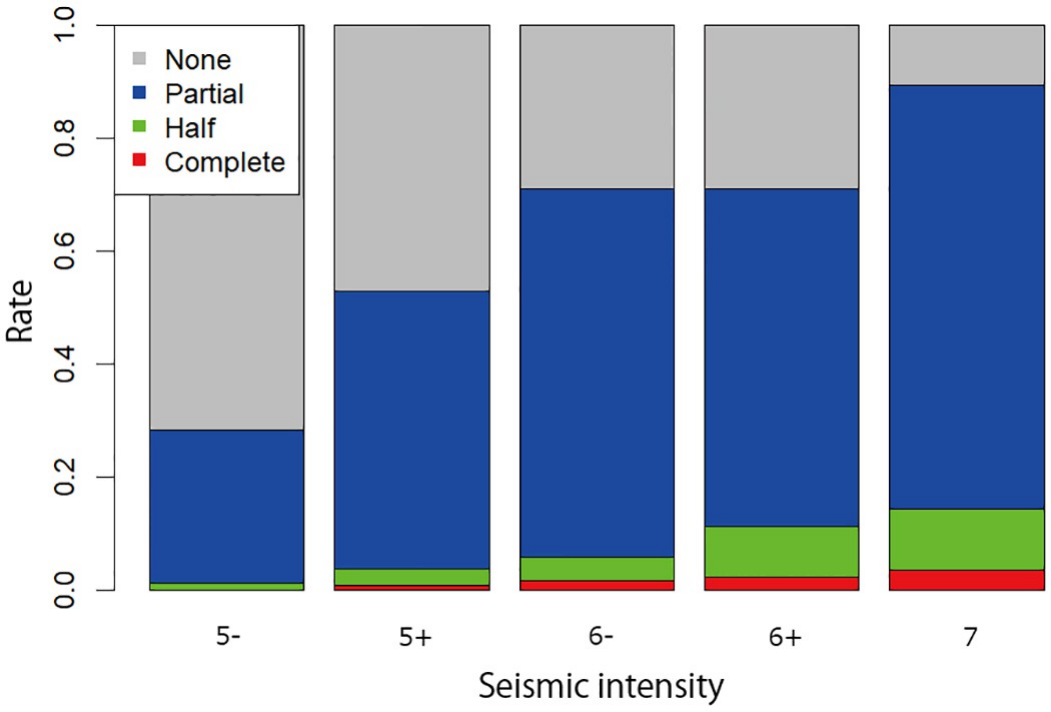
The relationship between the seismic intensity and the proportion of establishments with various levels of damage. The gray, blue, green, and red parts of each bar show the proportion of establishments with no, partial, half, and complete damage to their production facilities, respectively, as taken from the RIETI survey, depending on the seismic intensity (5-, 5+, 6-, 6+, and 7) taken from [23].

**Fig 2:**
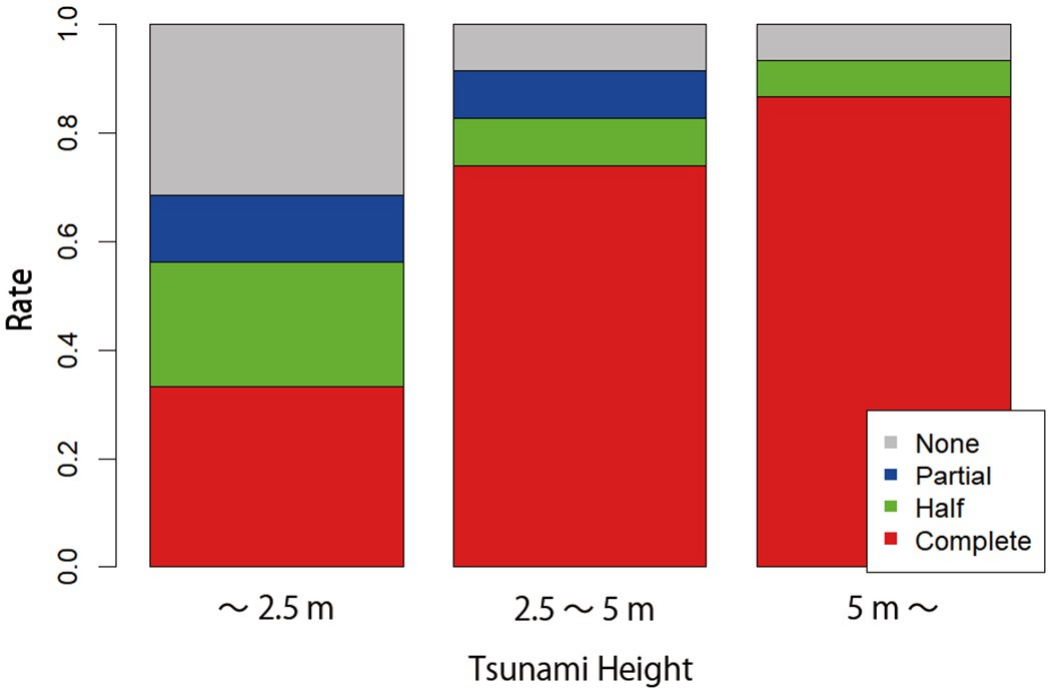
The relationship between the tsunami height and the proportion of establishments with various levels of damage. The gray, blue, green, and red parts of each bar show the proportion of establishments with no, partial, half, and complete damage to their production facilities, respectively, as taken from the RIETI survey, depending on the tsunami height (< 2.5*m*, 2.5 − 3*m*, *and* > 3*m*) taken from [24].

For this purpose, we first examine the correlation between the level of damage to each firm reported in the RIETI data and the intensity of the earthquake and the depth of the tsunami of the city where the firm is located, which are taken from the seismological and tsunami data. Figs 1 and 2 show how the earthquake intensity and the tsunami height are correlated with the level of damage at the firm level. By using the probability of each level of damage to a firm given an earthquake intensity and tsunami height, we randomly assign the level of the damage to each establishment not covered in the RIETI data but included in the TSR-Census data. If an establishment is affected by both the earthquake and the tsunami, the level of its damage is determined by the heavier damage caused by either the earthquake or tsunami. For example, if the estimated level of damage to an establishment by the earthquake is “half destroyed” while that by the tsunami is “completely destroyed,” we assume that production facilities of the establishment were completely destroyed. Similarly, we use the relationship between the tsunami height from the tsunami data and the duration of a power outage from the RIETI data to estimate the duration of power outage for each establishment included in the TSR-Census data.

#### Data on actual production after the GEJE

Finally, we need data for production in frequent intervals, rather than the yearly or quarterly gross domestic product (GDP) of Japan, to obtain parameter values that provide a better fit between the simulated and actual production. For this purpose, we utilize the Indices of Industrial Production (IIPs) [25] that indicate the monthly production in the manufacturing sector as a percentage of its average in a particular month and year. We use the IIPs, not the indices of all production activity (IAPA), because the IAPAs are not available at the prefecture level. Therefore, we assume that the rate of reductions in production of non-manufacturing sectors was the same as that of the manufacturing sector. By using the yearly GDP and monthly IIPs, we construct the total monthly value-added production of Japanese firms.

### Model

#### Overview and key assumptions

We employ the dynamic ABM at the firm level of [13–15], which is an extension of the model of [26]. Although our simulation incorporates establishment-level data in addition to firm-level data, as explained in the previous section, we use establishment-level data mainly to estimate damage to production facilities in disaster-hit regions. Therefore, our model is at the firm level, although the initial shock is given at the establishment level.

Each firm utilizes a fixed amount of labor and various intermediates provided by its suppliers, produces its product, and sells the product to client firms and the final consumers. Supply chains are a priori determined by the data and fixed over time: Even after an economic shock, such as a natural disaster, firms cannot replace disrupted suppliers or clients with new ones.

We assume a Leontief production function where factors of production, i.e., certain types of intermediate goods, labor, and electricity, are required in fixed proportions predetermined by the data. Products are industry specific, and thus firms in the same industry produce the same product. Industries are defined by the Japan Standard Industrial Classification [19]. Firms hold an inventory of each intermediate good to prepare for a shortage of supplies, although no inventory of service inputs or produced goods within the producer firms is assumed.

Following standard ABMs, our model does not assume the profit maximization of firms but assumes several simple rules for demand and supply, as explained in detail later. In the initial period without any shock to supply chains, or on day 0, the demand for and supply from any firm are equal to each other. At the end of day 0, a natural disaster hits some regions in the economy, and hence, production facilities in the affected regions are damaged. In addition, the supply of electricity in the affected regions is limited for a certain period after the disaster. Accordingly, the production capacity of firms affected by the disaster and power outages declines, and thus, they may have to ration their products to their suppliers and consumers. Reductions in production propagate upstream and downstream through supply chains because firms directly affected by the disaster reduce demand for inputs from their suppliers and the supply to their clients.

**Fig 3:**
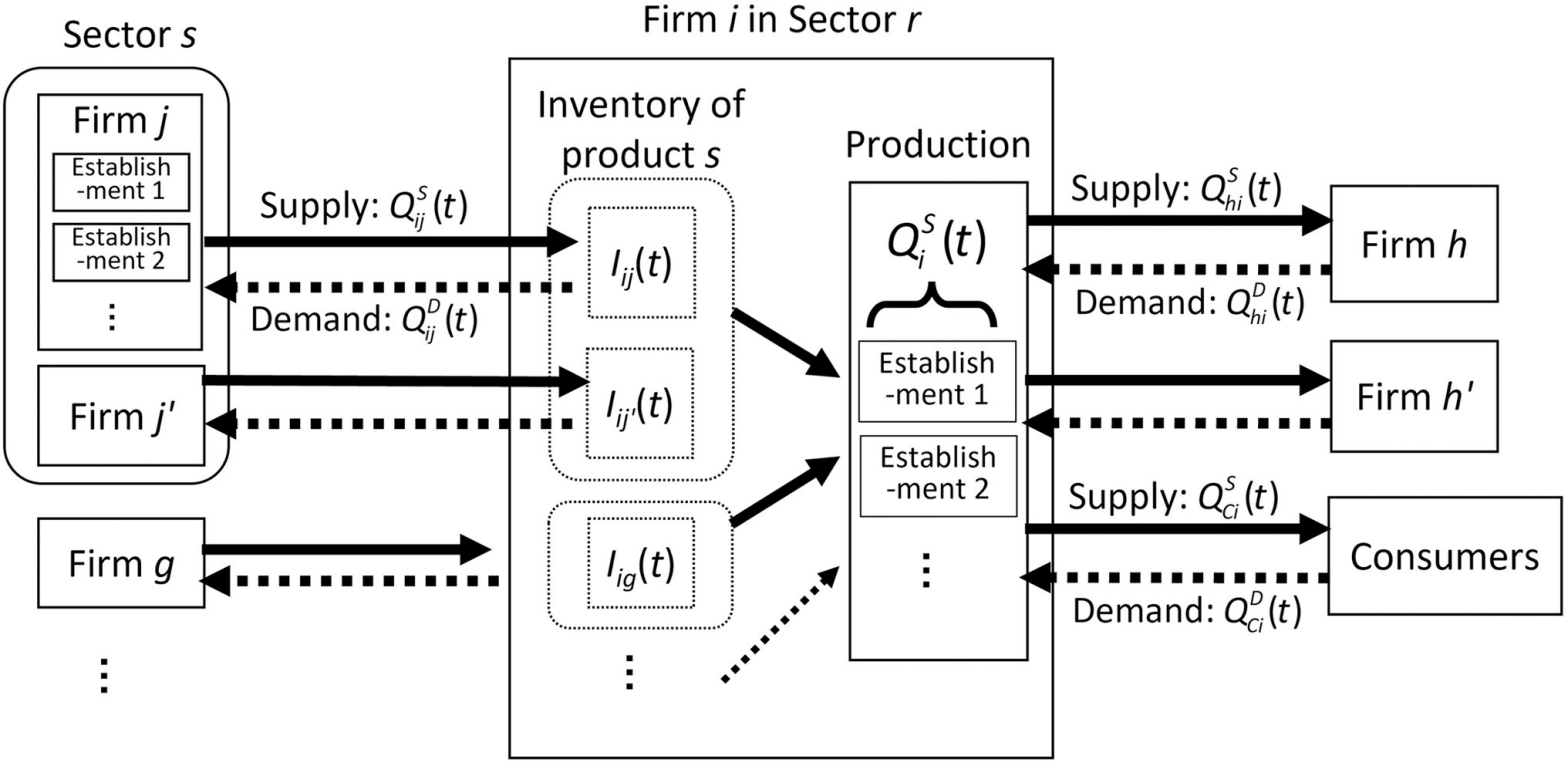
Overview of the agent-based model. The supply of products flows from the left to the right, whereas demand flows in the opposite direction.

An overview of the model is depicted in Fig 3. The source code to execute the model is on GitHub [27], as is the correspondence between the code and the model.

#### Demand and supply in the predisaster period

We start with the description of the economy without any supply chain disruption on day 0. In the following, the supply of the intermediate product from supplier *i* to client *h* on day *t* is denoted by $Q_{hi}^{S}\left(t\right)$, and the supply of firm *i* to the final consumers is denoted by $Q_{Ci}^{S}\left(t\right)$. Then, the production of firm *i* on day 0 is given by

$$\begin{array}{c} Q_{i}^{S}\left(0\right)=\Sigma_{h}Q_{hi}^{S}\left(0\right)+Q_{Ci}^{S}\left(0\right). \end{array}$$ (1)

Demand for products is determined in the following two ways. First, firms predict that the demand for their product is the same as that on the previous day, $Q_{i}^{D}\left(t-1\right)$. Therefore, firm *i* demands supplier *j*’s product of an amount $Q_{ij}^{S}\left(0\right)Q_{i}^{D}\left(t-1\right)/Q_{i}^{S}\left(0\right)$. Second, firms demand intermediates to stock inventories. We denote firm *i*’s inventory of the intermediate produced by firm *j* at the beginning of day *t* by *I*_*ij*_(*t*). Firm *i* aims to restore this inventory to a level $n_{i}Q_{ij}^{S}\left(0\right)$ so that supplies for *n*_*i*_ days are stocked. We assume that *n*_*i*_ is randomly determined by a Poisson distribution where the mean is *n*. When the actual inventory is smaller than its target, firm *i* increases its inventory gradually by 1/*τ* of the difference day by day, where *τ* = 6 following [28]. Accordingly, firm *i*’s demand for the product of its supplier *j* on day *t*, $Q_{ij}^{D}\left(t\right)$, is the sum of the demand for production and inventory:

$$\begin{array}{c} Q_{ij}^{D}\left(t\right)=Q_{ij}^{S}\left(0\right)\frac{Q_{i}^{D}\left(t-1\right)}{Q_{i}^{S}\left(0\right)}+\frac{1}{\tau }[n_{i}Q_{ij}^{S}\left(0\right)-I_{ij}\left(t\right)]. \end{array}$$ (2)

By summing the demand from all clients and the final consumers, we obtain the total demand for the product of supplier *i* on day *t*, $Q_{i}^{D}\left(t\right)$:

$$\begin{array}{c} Q_{i}^{D}\left(t\right)=\Sigma_{h}Q_{hi}^{D}\left(t\right)+Q_{Ci}^{D}. \end{array}$$ (3)

On day 0, we assume that the level of inventory is equal to its target level ($n_{i}Q_{ij}^{S}\left(0\right)=I_{ij}\left(0\right)$) and that the demand for the product of firm *i* on the previous day is equal to its production ($Q_{i}^{D}\left(-1\right)=Q_{i}^{S}\left(0\right)$). Therefore, there is no excess supply or demand on day 0: $Q_{ij}^{S}\left(0\right)=Q_{ij}^{D}\left(0\right)$ and $Q_{i}^{S}\left(0\right)=Q_{i}^{D}\left(0\right)$.

#### Reduction in production capacity because of a natural disaster

We suppose that a natural disaster hits some regions of the economy at the end of day 0. The disaster shrinks the production of firms in two ways. First, because the disaster caused the destruction of production facilities and power outages of establishments in the affected regions, their production capacity declined. When any establishment of a firm is affected by the disaster, production of the firm declines by the amount estimated from the share of the establishment in the predisaster production of the firm.

In particular, we assume that establishments in the power outage regions shut down their production. The duration of a power outage for establishments is reported in the RIETI data if they are surveyed; otherwise, they are estimated from the tsunami height. In addition, although the reported duration is more than six months for some establishments, we assume that establishments in the power outage regions recover their production fully *ξ* days after the disaster. The value of *ξ* is to be calibrated. This assumption is because when the duration of the power outage is very long, suppliers and clients connected to establishments experiencing a power outage may find other partners and recover production. Instead of modeling the dynamics in the supply chains, we simply assume the maximum duration of the power outage.

After (re)starting operation in the post-disaster period, firms’ production capacity is lower than in the predisaster period. More precisely, the rate of reduction in the production capacity of firm *i* in the disaster regions on day *t*, *δ*_*i*_(*t*), is determined by a larger value among the reduction rates because of the damage to production facilities, $\delta_{i}^{\text{f}}\left(t\right)$, and that because of power outages, $\delta_{i}^{\text{p}}\left(t\right)$:

$$\begin{array}{c} \delta_{i}\left(t\right)=\text{max}\left(\delta_{i}^{\text{f}}\left(t\right),\delta_{i}^{\text{p}}\left(t\right)\right). \end{array}$$ (4)

More specifically, $\delta_{i}^{\text{f}}\left(1\right)$ is determined by the level of damage reported by the firm (establishment) taken from the RIETI survey and the seismological and tsunami data, i.e., $\delta_{i}^{\text{f}}\left(1\right)$ is probabilistically determined by the location of firm (establishment) *i* at the city level and the probability of each level of damage in that city given by the RIETI data, the intensity of the earthquake, and the height of the tsunami. After that, damaged production facilities recover gradually. We assume that the rate of reduction in production capacity declines at the rate of *γ* with a damping factor *ζ*(*t*) equal to the ratio of healthy neighboring firms to all neighbors on day *t*. The damping factor corresponds to the peer effects observed in empirical studies [22]. Then, $\delta_{i}^{\text{f}}\left(t\right)$ is expressed as follows:

$$\begin{array}{c} \delta_{i}^{\text{f}}\left(t\right)=\left(1-\zeta \left(t\right)\gamma \right)\delta_{i}^{\text{f}}\left(t-1\right). \end{array}$$ (5)

In contrast, $\delta_{i}^{\text{p}}\left(t\right)$ is determined differently because the firms’ production reduction due to power outages does not recover gradually but recovers immediately and fully when electricity returns. Moreover, when firms are under a power outage, we assume that the firms can partly run their operation with a reduction rate of λ. Therefore, the rate of reduction in production because of the power outage for firm *i* in areas with power outage, $\delta_{i}^{\text{p}}\left(t\right)$, is defined as follows:

$$\begin{array}{c} \delta_{i}^{\text{p}}\left(t\right)=\{\begin{array}{cc} \lambda & \left(t\leq \xi \right)\\ 0 & (t>\xi ). \end{array} \end{array}$$ (6)

Given the two types of rates of reduction in production (Eqs 5 and 6), the larger one is chosen as the actual reduction rate, as shown in Eq 4. Accordingly, the maximum possible production of firm *i* on day *t*(≥ 1), ${\bar{Q}}_{i}^{S}\left(t\right)$, after the disaster is given by

$$\begin{array}{c} {\bar{Q}}_{i}^{S}\left(t\right)=Q_{i}^{S}\left(0\right)\left(1-\delta_{i}\left(t\right)\right). \end{array}$$ (7)

Second, the production of firm *i* may also be restricted by shortages of supplies from suppliers affected by the disaster. When facing a shortage of supplies from supplier *j*, firm *i* attempts to mitigate it by using its inventory of the supplies and by purchasing more from other existing suppliers in the same industry. In other words, we assume that inputs produced in the same industry are identical and substitutable to each other, although how much firms can substitute inputs from other existing suppliers for disrupted inputs depends on the production capacity of other existing suppliers. This assumption regarding input substitution may be justified because there are 1,460 industries in our simulation. Then, the maximum possible production of firm *i* limited by the shortage of supplies from industry *s* is:

$$\begin{array}{c} {\bar{\bar{Q}}}_{i(s)}^{S}\left(t\right)=\frac{\Sigma_{j\in s}I_{ij}\left(t\right)}{\Sigma_{j\in s}Q_{ij}^{S}\left(0\right)}Q_{i}^{S}\left(0\right). \end{array}$$ (8)

The two sources determine the maximum possible production:

$$\begin{array}{c} Q_{\text{max}i}^{S}\left(t\right)=\text{Min}({\bar{Q}}_{i}^{S}\left(t\right),{\text{Min}}_{s}\left({\bar{\bar{Q}}}_{i(s)}^{S}\left(t\right)\right)). \end{array}$$ (9)

Therefore, the actual supply of firm *i* on day *t* is either determined by the maximum possible production or the demand:

$$\begin{array}{c} Q_{i}^{S}\left(t\right)=\text{Min}(Q_{\text{max}i}^{S}\left(t\right),Q_{i}^{D}\left(t\right)). \end{array}$$ (10)

#### Demand and supply after the disaster

When the demand for firm *i*’s product surpasses its production capacity after the disaster, the firm rations its product to its client firms and the final consumers because this model does not assume price adjustment, following some simple rules explained in detail in S1 Appendix. In brief, in this rationing process, any of the clients and the final consumers can obtain a positive amount of the production, whereas clients that demand less after the disaster relative to the predisaster demand can meet a larger portion of their demand.

Once the rationing rules determine the supply for each client, the inventory of firm *j*’s product held by firm *i* on day *t* + 1 is updated:

$$\begin{array}{c} I_{ij}\left(t+1\right)=I_{ij}\left(t\right)+Q_{ij}^{S}\left(t\right)-Q_{ij}^{S}\left(0\right)\frac{Q_{i}^{D}\left(t-1\right)}{Q_{i}^{S}\left(0\right)}. \end{array}$$ (11)

This equation, combined with Eqs (2) and (10), determines the demand of firm *i* for the intermediate good supplied by firm *j* on day *t* + 1, $Q_{ij}^{D}\left(t+1\right)$, and the total demand for firm *i*’s product $Q_{i}^{D}\left(t+1\right)$. The supply of firm *i* on day *t* + 1, $Q_{i}^{S}\left(t+1\right)$, is then determined by Eqs (7)–(10).

### Simulations

#### Full model

The GEJE is a mega earthquake that hit the northeastern part of Japan on March 11, 2011, causing massive human and economic losses, including approximately 15 thousand deaths and a loss of economic stocks (social infrastructure, houses, and facilities) of 16.9 trillion yen [29]. To simulate how the effect of the GEJE on production propagated through supply chains, we calibrate the model by using the data described in the previous sections and then estimate the parameter values in the model by using the following optimization process.

First, our model includes the following parameters to be calibrated: the recovery rate of production capacity after the GEJE, *γ*; the maximum duration of the power outage in days, *ξ*; the mean of the targeted size of the inventory of intermediate products from suppliers measured by how many days of intermediate production are stored, *n*; and the reduction rate of production capacity because of the power outage after the GEJE, λ. We further assume that the recovery rate and the mean target inventory size are sector specific and vary across three sectors, i.e., the primary, the manufacturing, and the service sectors, for the following reasons. First, because the primary and service sectors rely less on production facilities than the manufacturing sector, the recovery of the former two sectors may be quicker than that of the latter. Second, inventory turnover varies depending on the characteristics of products and markets. For example, it tends to be high for firms in the service sector, such as retailers, because of low margins and low for firms in the light manufacturing industry because of low holding cost [30]. In our simulation, we experiment with a recovery rate ranging from 0.01 to 0.30 with an interval of 0.01. Similarly, we use the range and interval of experimental values of other parameters as specified in Table 1. Therefore, there are eight parameters to be calculated. Accordingly, the parameter space of the model with the full set of parameters is 6.7 × 10^11^.

**Table 1:** Parameter values obtained from calibration.

Parameter	Definition	Range	Interval
*γ*	Recovery rate of production capacity (sector specific)	0.01–0.30	0.01
*n*	Mean target inventory size (days, sector specific)	4–30	1
*ξ*	Maximum duration of power outages (days)	4–30	1
λ	Reduction rate of production capacity because of power outages	0.10–1.00	0.01

The initial state of each simulation run is randomly determined in the following two ways. First, the level of damage to production facilities of each establishment not included in the RIETI data; in the disaster regions, the level of damage to a facility is randomly determined given its location and the intensity of the GEJE and the depth of the tsunami at the city level. Second, the target inventory size of each firm is randomly determined by the Poisson distribution with mean *n*. We select three different initial states randomly and run the simulation three times for each set of parameter values. More trials with different initial states can possibly provide a more reliable fit of the model because the variance in production from the different initial states is large. However, because the parameter space is so large that a simulation run with a particular set of parameter values takes approximately several hours, we experiment with only three initial states in the optimization process.

By using a set of parameter values and three initial states, we simulate production dynamics over the three runs for 365 days after the GEJE. Then, we compute the mean squared error (MSE) between the daily simulated and actual production. Because we rely on the monthly IIPs for the actual production, we define daily production from the IIPs and GDP simply assuming that the IIP of a month can be applied to any day in the month. By using the actual daily production, we can calibrate the model so that the MSE is minimized. We note that in the calibration, it is too time-consuming to employ random or grid search in the optimization process because the parameter space is extremely large. Therefore, we use Bayesian optimization so that the parameter search is conducted more efficiently.

#### Simplified models

In addition to the benchmark model with the full set of parameters, or the full model, we simulate two simplified models to examine what factors lead to a better fit. First, we experiment with a model with only three parameters, i.e., *γ*, *ξ*, and *n*, following [13]. Any of these three is not assumed to be sector specific. By comparing the result from the simplified model with that from the full model, we can examine whether and how much adding parameters improves the fit between the actual and simulated production after the GEJE. Second, we simulate another simplified model assuming no power outages after the GEJE to examine how incorporating power outages into the simulation affects the predictive power of the model. In other words, we assume λ = 0 and *ξ* = 0 and thus drop these two parameters from our simulation. The range and interval of each of the parameters used in the simplified models are the same as those used in the full model.

## Results

### Optimization process

**Fig 4:**
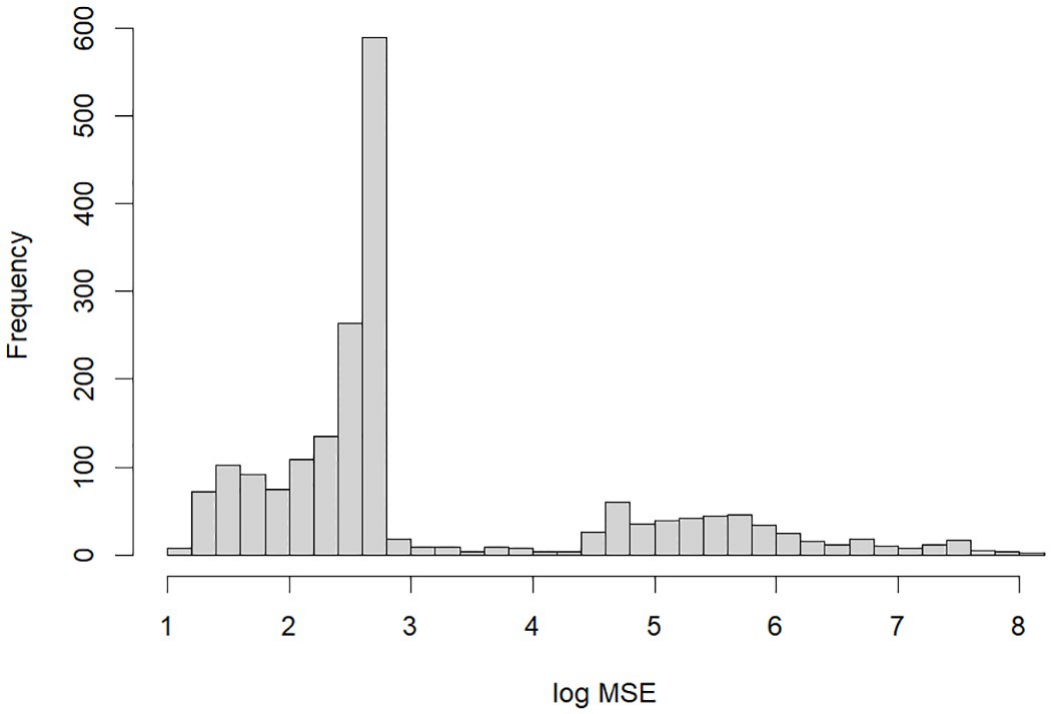
The distribution of the mean squared error in log scale from the optimization process using the full model.

Fig 4 shows the distribution of the MSE in log scale from the optimization process to search for the best parameter values using the full model. The number of sets of parameter values searched is 1,957. In the optimization process, we first experiment with 100 sets of parameter values selected randomly. In this first step, the distribution of the MSE is relatively flat because of the randomness, as indicated on the right side of Fig 4. After the first step, parameter values are chosen by an optimization algorithm in which the parameter space that generates a larger MSE than a threshold value is dropped from further experiments. As a result, there is a large gap in counts at the log MSE of approximately 2.8 in Fig 4. Moreover, counts shrink as the log MSE decreases on the left side of the figure, implying that the optimization process gradually decreases the MSE and finally obtains parameter values that are close to optimal.

**Fig 5:**
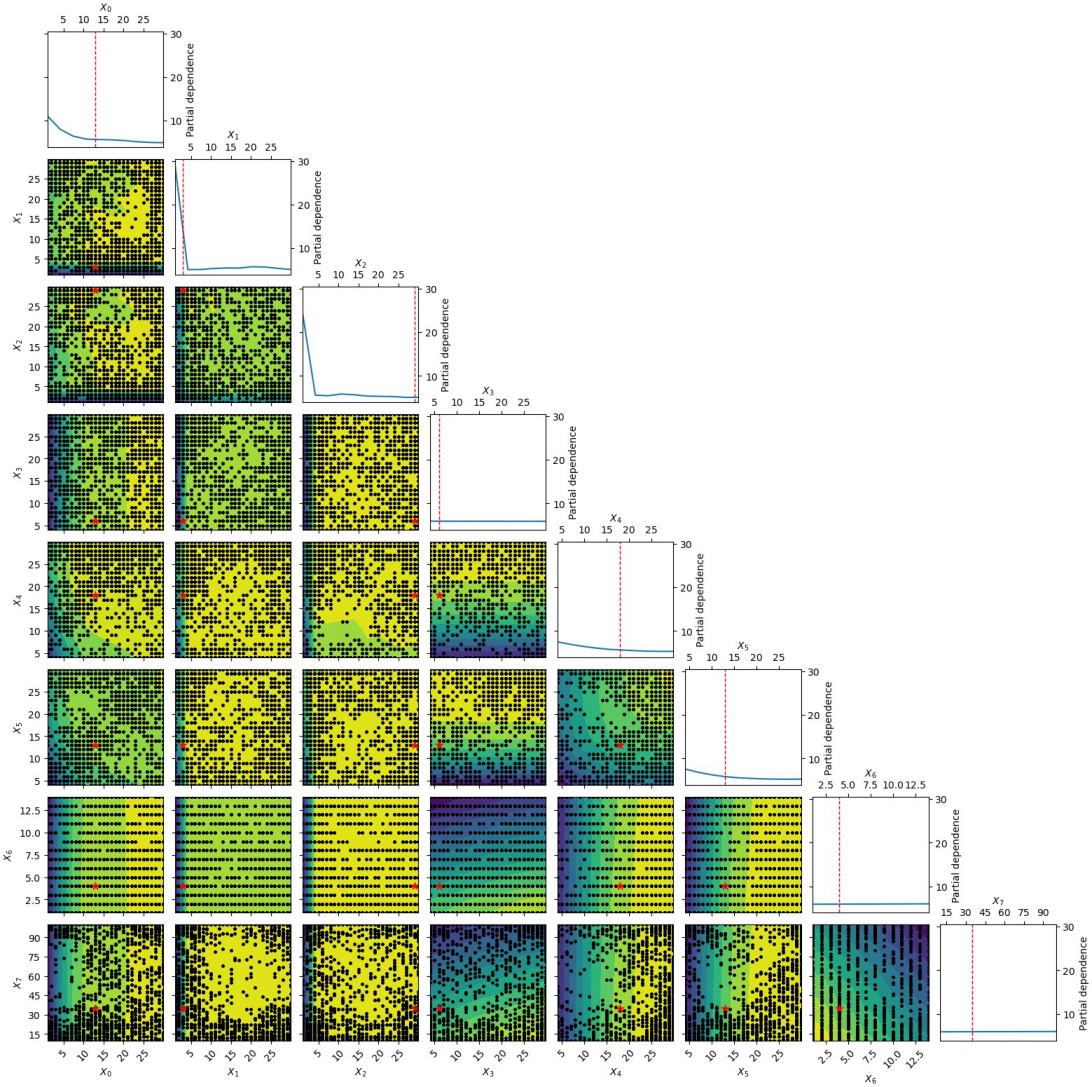
The mean squared error (MSE) distribution from simulations using the full model and assuming different set of parameter values. Parameters x0-x6 indicate the recovery rate (%) for the primary, secondary and service sectors, the inventory size for the primary, secondary and service sectors, the power outage cutoff day, and the power outage loss rate (%), respectively. The figures with black dots and yellow and green areas are contour graphs. Each block dot in a contour graph represents an MSE assuming the values of the two parameters shown on the two axes and the averages of all other five parameters. Yellow and green areas indicate smaller and larger MSEs, respectively, from the simulations. Each of the white diagonal figures with red and blue lines presents the distribution of the MSEs (partial dependence) from simulations, assuming a particular value of the parameter shown at the top of the figure and averages of all other six parameters. Each red star in a contour graph is the set of two parameter values that generate the smallest MSE.

Fig 5 displays the optimization process when using the full model. More precisely, the diagonal panels in the figure show the distribution of the root MSE based on surrogate models where the value of a particular parameter changes while other parameters are averaged out. For example, the top panel indicates that the error decreases as *x*_0_, or the recovery rate (%) for the primary sector increases. Further assuming that the values of two parameters change while others are averaged out, the off-diagonal panels show contour graphs that indicate the MSE distribution by color (yellow and green areas indicate small and large MSEs, respectively) and plots of parameter values used for simulation in black.

From the optimization process, we find that the best parameter values are 0.13 for the recovery rate of the primary sector, 0.03 for that of the manufacturing sector, 0.29 for that of the service sector, 6 for the mean target inventory size of the primary sector, 18 for that of the manufacturing sector, 13 for that of the service sector, 0.35 for the power outage loss rate, and 4 for the power outage truncate duration (days). These optimized values are shown by red lines in the diagonal panels of Fig 5 and red dots in the off-diagonal panels.

Several findings in the optimization process should be noted. First, the MSE distribution when we change the inventory size for the primary sector (*x*_3_) looks almost flat, suggesting that this parameter does not substantially affect the MSE. Because the primary sector uses fewer inputs than that of the other sectors, the size of the intermediate inventory may negligibly affect the simulation results. Second, the distribution of the MSE when changing the power outage loss rate (*x*_6_) and the power outage truncate duration (*x*_7_) is almost flat. This finding implies that the value of the two parameters does not substantially affect the simulation results. We will discuss these issues in detail in the next section.

### Comparing predictions between the full and simplified models

**Fig 6:**
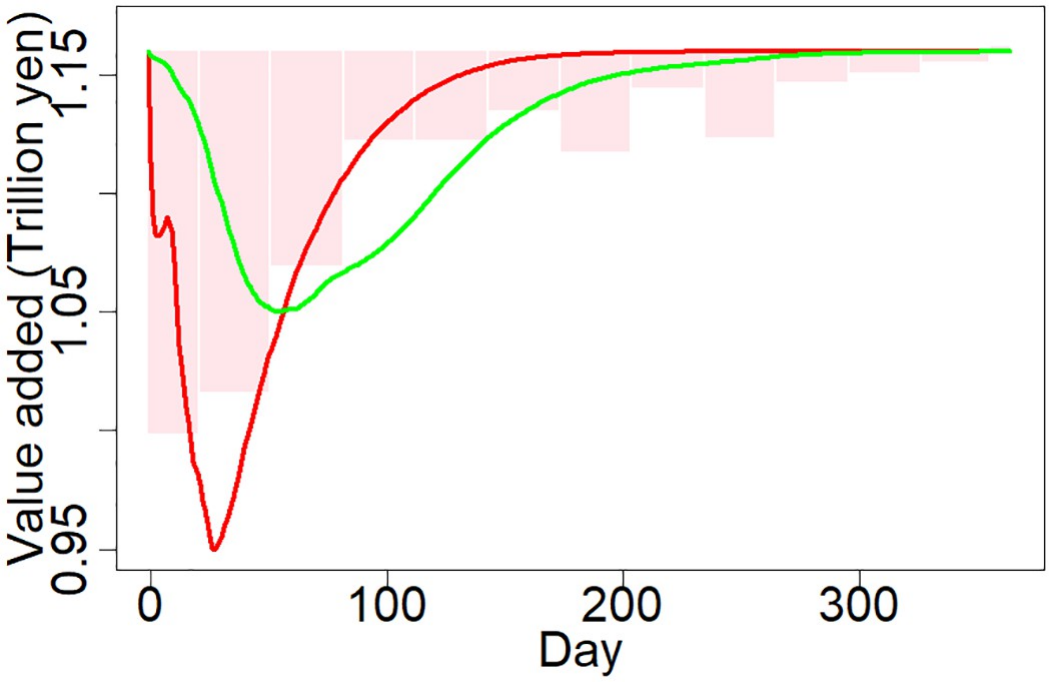
Simulation result and comparison with the previous study. The red and green lines show the average daily value added production after the GEJE from the 30 simulations of this study and the previous study [13], respectively. The pink bars represent the production loss estimated from the IIPs. The horizontal axis shows the number of days after the GEJE, whereas the vertical axis indicates the daily value added production.

Fig 6 shows the simulation results. The red line presents changes in the daily total value added production of Japanese firms for 365 days after the GEJE when using the model with the full set of eight parameters and the parameter values that lead to the best fit with the actual production. The daily value added is averaged over the three runs with different initial states. The green line presents the corresponding changes in value added when using the simple model with only three parameters and firm-level data of [13]. The pink bars indicate the actual production estimated from the monthly IIPs and GDP. The MSEs between the simulated and actual daily production for the full and simple models are 3.24 and 15.4 (their square roots are 1.80 and 3.93), respectively.

This finding suggests that the model with eight parameters can predict the actual production dynamics after the GEJE more precisely than the simple model with three parameters. In particular, Fig 6 shows that the full model can explain the large reduction in production immediately after the GEJE, possibly because the simulation of the full model incorporates damage to production facilities of non-headquarters establishments and the power outage in the disaster-hit regions, thus appropriately estimating the propagation of the shock from the disaster regions to other regions.

**Fig 7:**
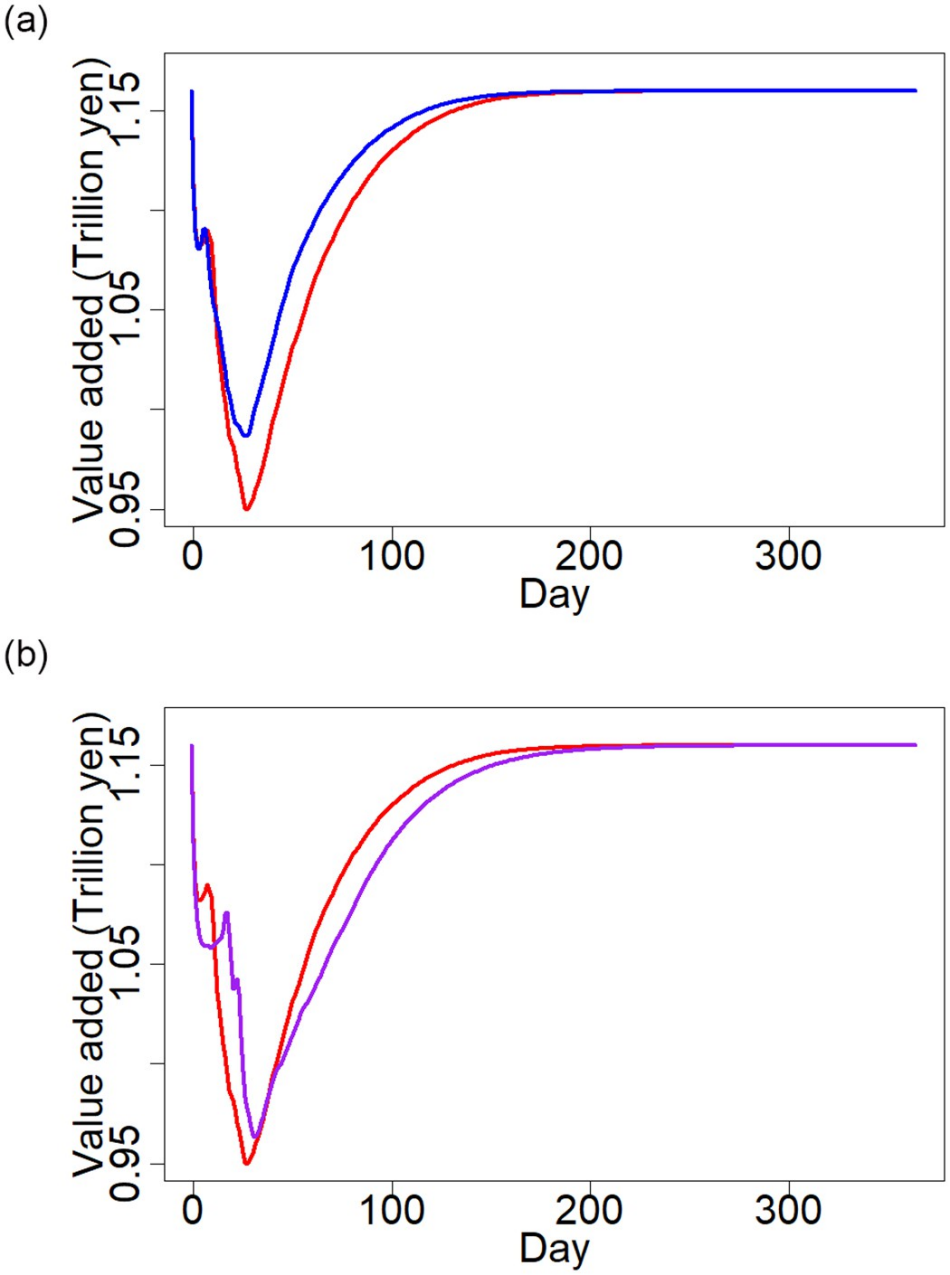
Comparisons between simulation results from the full and the two simplified models. In Panel (a), the red and blue lines indicate the average daily value added production from the 30 simulations using the full model and the simplified model assuming no power outage, respectively. In Panel (b), the red and purple lines indicate the daily value added production from the simulation using the full model and the simplified model with only three parameters, respectively. The horizontal axis shows the number of days after the GEJE, whereas the vertical axis indicates the daily value added production.

Fig 7 shows a comparison of the production dynamics predicted by the full model and by other simplified models defined earlier. Panel (a) shows the comparison with the model assuming no power outage. The gap between the production dynamics from the two models indicates the production loss because of the power outage. In total, the difference between the total production predicted by the two models is 2.7 trillion yen, or 11.8% of the production loss predicted by the full model (11.8 trillion yen).

Panel (b) shows the comparison with the model with only three parameters, i.e., the recovery rate, the recovery delay, and the mean target inventory size. In addition, we assume that these parameters are not sector specific; that is, all firms share the same values of the three parameters. Although this model is similar to that used in the prior study [13], presented by the green line in Fig 6, in this model, we incorporate initial shocks at the establishment level rather than at the firm level used in [13]. The MSE given by the best solution when using the model with three parameters is 3.55 (the square root is 1.88), compared with 3.24 generated by the full model. Therefore, the difference between the predictions of the two models is not quite large.

## Discussion and conclusions

In this paper, we simulate the economic loss because of supply chain disruptions triggered by the Great East Japan earthquake (GEJE) in 2011. We particularly improve the data and model used in previous studies [13, 14] by incorporating more parameters into the model and by employing various data sources, such as establishment-level census data, post-disaster survey data, and seismological and tsunami data.

The full model with eight parameters that uses the newly constructed data successfully improves the fit between the simulated and actual production after the GEJE compared with that of the previous model in [13, 14], as shown in Fig 6, for the following four possible reasons. First, we estimate the initial economic shock of the GEJE more accurately from the new data by identifying non-headquarters establishments in the disaster-hit regions and the level of damage to production facilities and the duration of production shutdowns in each district. Second, we incorporate the negative effect of the post-disaster power outages on production that was quite substantial for a few weeks after the GEJE. Third, because we can identify indirect supply chain linkages between establishments through their headquarters by using establishment-level data on firm attributes and firm-level data on supply chains, the simulation in this study replicates propagation of the economic shock through supply chains more accurately. In particular, without information on supply chains between establishments, the initial propagation from non-headquarters establishments in the disaster regions to others may be heavily undervalued. Finally, assuming more parameters are calibrated provides us with more flexibility so that the predicted outcome can be closer to the actual outcome.

Among the four potential reasons, the last one, a larger number of parameters, does not seem to result in a large improvement in the predictive power because Panel (b) of Fig 7 clearly shows that the predictions from the models with eight and three parameters are quite similar when they use the same detailed data. This finding may imply that the model with the three parameters can sufficiently explain the propagation of economic shocks through supply chains, whereas the poor fit found in [13] relative to the fit from the full model (Fig 6) can be attributed to the first factor, limitations of the data in the former. In contrast, we find that the second factor, i.e., the incorporation of power outages, improves the predictive power to a certain extent, as shown in Panel (a) of Fig 7. However, the size of the improvement is not very large, accounting for 11.8% of the total production loss. Therefore, we conclude that the improvement in the predictive power by our new simulation method is mostly due to our detailed data at the establishment level.

Although this paper focuses on an investigation of how our extended simulations using new data and method can predict the effect of the GEJE more precisely, our simulation can be applied in various ways. First, using the simulation, we can predict the economic effect of future disasters, such as the Nankai Trough earthquake, on each region more accurately. Although this investigation has been conducted in [31] using a CGE model, it is interesting to examine how the result from our firm-level agent-based approach is different from that from the region-industry-level CGE approach. In addition, our simulation approach enables us to explore how network topology affects the propagation of economic shocks through firm-level supply chains, as in some previous studies [13, 14].

Finally, we mention several remarks from this study. First, we discussed just above a possible reason for the small improvement by adding parameters in the full model. An alternative reason for this is that we failed to find the optimal parameter values in our optimization process because the parameter space for the full model is substantially larger (6.7 × 10^11^) than that of the model with three parameters. Conducting more experiments for further optimization may lead to a better fit.

**Fig 8:**
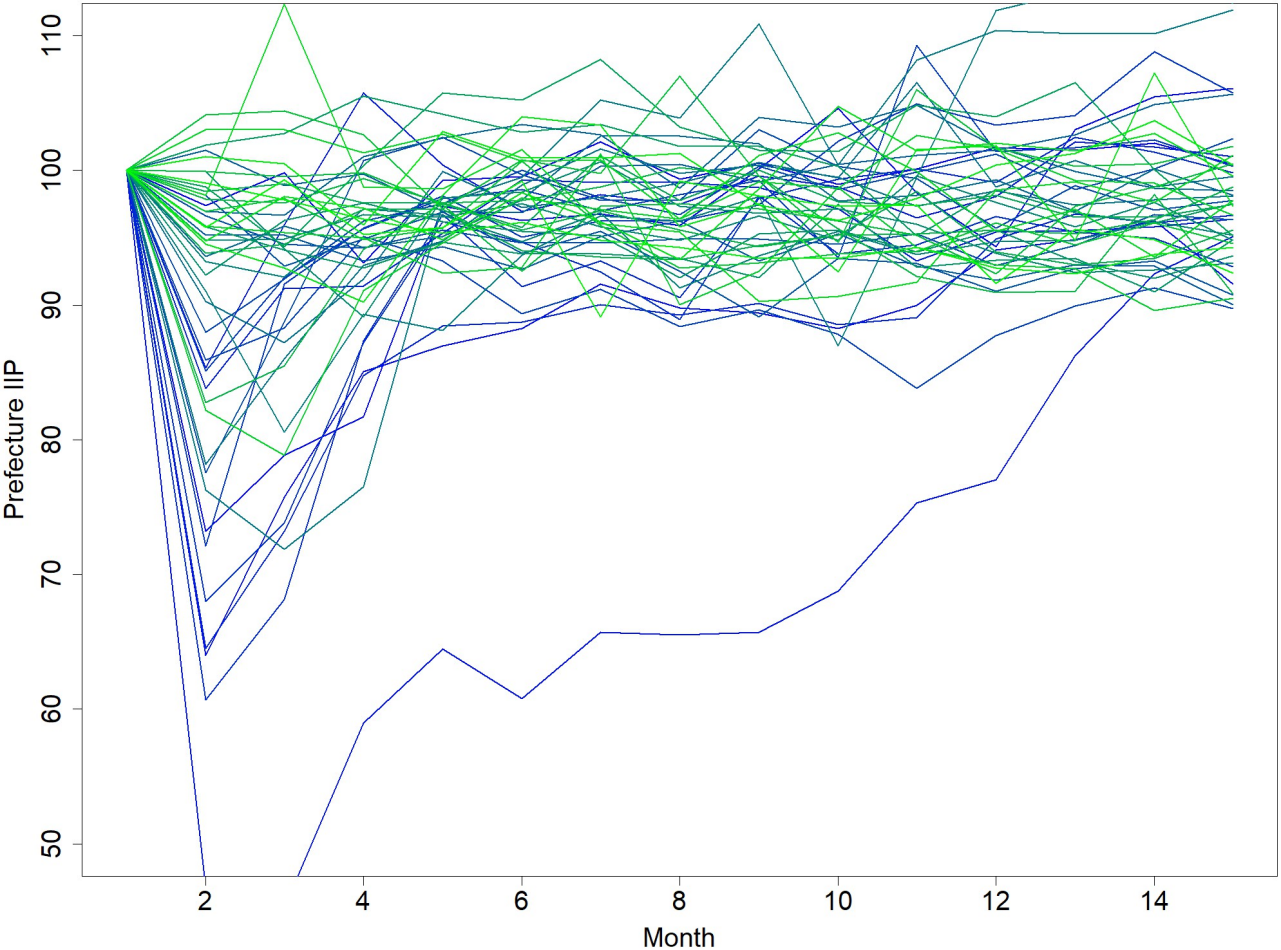
Changes in the indices of industrial production (IIPs) over time at the prefecture level. Each blue and green line in this figure shows the IIPs of a particular prefecture in and out of the disaster regions, respectively, during the 15-month period after the GEJE. The IIP of any prefecture on the day of the GEJE is normalized to be 100.

Second, although we fit the predicted value with the total production of Japan in the optimization process, it is also possible to fit the predicted and actual values of value-added production of each prefecture because the IIPs are available at the prefecture level (Fig 8). However, when we attempted to do so, we found that the difference between the predicted and actual production at the prefecture level was quite large and that the sum of the differences for all prefectures is larger than the corresponding difference when we fit the predicted and actual total production of Japan. These findings contradict the standard consideration that more observations help to obtain a better fit. If we can improve the model, we might be able to obtain a better fit by utilizing production at the prefecture level.

Third, the distribution of the MSE when changing the value of the loss ratio and duration of power outage (*x*_6_ and *x*_7_ in Fig 5) is almost flat. This indicates that the values of the two parameters may not be accurately estimated because any change in the parameter value does not improve the fit substantially, and if anything, different values could lead to a better fit. The difficulty in determining the optimal value of the two parameters can lead to a bias in the simulation.

Fourth, we admit that we ignore some heterogeneity among establishments and among supply chain links. For example, when firms suffer from the same level of damage to their production facilities because of disasters, the rate of reduction in the production capacity can vary depending on firms’ preparedness for risks of supply chain disruptions and the strength of their links with suppliers and clients.

Finally, although we incorporate production reductions due to the power outage after the GEJE into our analysis, other factorsb that caused large reductions immediately after the GEJE, such as damage to water and gas pipes and transport infrastructure and labor shortages because of the evacuation from the disaster regions, are still ignored. This may lead to a poor fit between the actual and simulated production in the first month. We leave these issues for future research.

## Supporting information

S1 Appendix Rationing rules. (PDF)
