# Supplementary material for: Simulation of supply chain disruptions considering establishments and power outages
Source: PLoS One. 2023 Jul 7;18(7):e0288062. doi: 10.1371/journal.pone.0288062 (PMC10328253; doi:10.1371/journal.pone.0288062)
Supplement: S1 Appendix — (PDF) [file pone.0288062.s001.pdf]

Simulation of supply chain disruptions considering establishments and power outages

Supporting Information

Hiroyasu Inoue

Yoshihiro Okumura

Tetsuya Torayashiki

Yasuyuki Todo

Rationing Rules To explain the rationing rules, we denote the ratio of client j's demand for the product of firm i to its initial demand by $q^D_{ji}(t) \equiv Q^D_{ji}(t)/Q^S_{ji}(0)$ and the corresponding ratio for the demand of final consumers by $q^D_{Ci}(t) \equiv Q^D_{Ci}(t)/Q^S_{Ci}(0)$. Then, the supply to each client and each consumer is determined by the following steps. At the beginning of step x, the amount of production that has not been rationed and remains to be rationed is defined as $Q^R_i[x]$. We also define the minimum ratio of the current demand to the initial demand by $q^D_{\text{min}}(t) \equiv \text{Min}(q^D_{ji}(t), q^D_{Ci}(t))$. In the first step, where $x = 1$ and $Q^R_i[1] = Q^S_i(t)$ by definition, if

$$Q^R_i[x] \geq q^D_{\text{min}}(t)Q^D_i(t), \tag{1}$$

firm i rations to each client firm and consumer the amount of its demand multiplied by the minimum demand ration $q^D_{\text{min}}(t)$. The remaining production, $Q^R_i[x+1] = Q^R[x] - q^D_{\text{min}}(t)Q^D_i(t)$, is handed over to the second step. In the second step, a client firm or the aggregate consumer that satisfies its demand (or whose rate of the current demand to the initial demand is at the minimum) is dropped. In contrast, if Equation (1) does not hold in the first step, firm i rations to each client and consumer the amount of the demand multiplied by the ratio of the remaining production to demand defined by $q^D_{\text{r-d}_i} \equiv Q^R_i[x]/Q^D_i(t)$. Accordingly, the remaining production $Q^R_i[x+1]$ is equal to $Q^R_i[x] - q^D_{\text{r-d}_i}(t)Q^D_i(t)$ and handed over to the second step. In future steps, we repeat this procedure until $Q^R_i[x]$ becomes zero.
